# Presence of Porcine Circovirus Type 2 in the Environment of Farm Facilities without Pigs in Long Term-Vaccinated Farrow-to-Wean Farms

**DOI:** 10.3390/ani12243515

**Published:** 2022-12-13

**Authors:** Gonzalo López-Lorenzo, Alberto Prieto, Cynthia López-Novo, Pablo Díaz, Susana Remesar, Patrocinio Morrondo, Gonzalo Fernández, José Manuel Díaz-Cao

**Affiliations:** Department of Animal Pathology (INVESAGA Group), Faculty of Veterinary Sciences, Universidade de Santiago de Compostela, 27002 Lugo, Spain

**Keywords:** biosecurity, environmental sampling, porcine circovirus type 2 (PCV2), real-time PCR, swine

## Abstract

**Simple Summary:**

Porcine Circovirus Type 2 (PCV2) vaccination is a widespread measure that effectively reduces infection prevalence in swine farms. However, this tool has failed to eradicate the infection, probably because of the persistence of PCV2 in the environment of the farms. Thus, the aim of the present study was to evaluate the presence of PCV2 in different areas of swine farms to identify critical points which may act as possible viral reservoirs. Four farrow-to-wean long-term vaccinated farms were studied, sampling different surfaces from animal and non-animal areas and analyzing them by qPCR to detect and quantify the PCV2 load. The results show the near absence of PCV2 in animal areas; in contrast, the virus is frequently detected in offices, farm staff clothing and the farm perimeter. As PCV2 vaccination does not provide sterilizing immunity, the detected viral load is probably the result of low shedding from subclinically infected animals on the farm; thus, PCV2 would tend to accumulate in areas not cleaned and disinfected regularly. Nevertheless, an external source of PCV2 cannot be totally ruled out. This study shows the existence of potential critical points for PCV2 persistence in vaccinated farms, indicating the need of considering them for future eradication plans.

**Abstract:**

Vaccination against Porcine Circovirus Type 2 (PCV2) even over several years has proven as an insufficient measure to eradicate the infection from farms, possibly due to not producing sterilizing immunity. Viral persistence in the farm environment has been proposed as a possible cause of reinfection, and for that reason, the main objective of this study was to identify potential critical points where PCV2 could persist in farrow-to-wean farms which had been vaccinating piglets for years. Surface samples were collected from different farm facilities with and without animals and analyzed by qPCR to detect and quantify the viral load. Most of the samples taken in animal housing facilities tested negative (96.6%); however, PCV2 was more frequently detected in samples from the offices (37.5%), the farm staff (25%) and the perimeter (21%). These results indicate that PCV2 contamination is frequent in facilities despite the long-term use of vaccination programs. Therefore, PCV2 control programs should include more exhaustive cleaning and disinfection protocols in non-animal facilities, as well as the implementation of specific biosecurity measures in these areas to minimize the risk of PCV2 introduction from external sources.

## 1. Introduction

Porcine Circovirus Type 2 (PCV2) is considered one of the most important pathogens for swine production due to the productive and economic repercussions that its infection entails [1,2]. It can infect pigs from the weaning age to adult ones, showing a considerable variety of syndromes [3]. One of the main characteristics of this virus is its structure: its small size and the absence of an external envelope provide a high resistance to adverse conditions [4,5,6]. Thus, the possibility that PCV2 may remain in the farm environment for extended periods should be considered in the control and preventive measures.

Vaccination against PCV2 is a highly recommended preventive measure in swine production due to two main reasons. First, this virus is considered to be present in all swine farms [1]. Second, vaccination has shown great efficacy in preventing PCV2 Systemic Disease and in avoiding the losses associated with it, reproductive losses and the decrease in productive performance associated with subclinical infection. Therefore, since the introduction of PCV2 vaccines in the 2000s, their use has been very widespread and nowadays pigs that reach slaughter are vaccinated in most countries [7]. As a consequence of the high PCV2 vaccination level on piglets, gilts/sows, or even both during these past years, in some farms, a considerable proportion of pigs already reach slaughter without having been infected [8,9,10].

PCV2 vaccines do not produce sterilizing immunity, but the protection that they confer avoids the infection of vaccinated piglets during the weaning phase so that, at least in farrow-to-wean farms, an eventual eradication would be possible [11]. Nevertheless, this has not been achieved yet despite its widespread use. In an attempt to attain the sought-after eradication, Feng et al. (2014) launched a protocol of mass vaccination against PCV2 (sows, boars and gilts: three doses/animal/year; piglets: vaccinated at 3 and 7 weeks of age) for one year. The results showed that neither PCV2 viremia nor any serological response to this virus were detected; however, when vaccination ceased, the infection became evident again. Thus, these authors suggested that the virus had never been completely wiped out from the farm [12]. Another possibility for the reinfection of farms could be an external source of PCV2. In this sense, visitors such as commercial agents, veterinary practitioners, etc., who usually visit different farms on the same day may introduce the virus to an uninfected farm, so the areas where these visitors are received (main entrance of the farm, office) could be critical points for the entry of the infection. Although farm visitors typically wear conventional disposable coveralls and boot covers, several previous investigations have revealed that their clothes or footwear could become contaminated in PCV2-infected herds, and therefore they may act as a “Trojan horse” for other farms [13,14,15]. Nevertheless, Patterson et al. (2011) managed to maintain a breeding herd free of infection for at least 20 months after using PCV2-naïve pigs for repopulating and performing an exhaustive cleaning and disinfection procedure together with a strict biosecurity plan [16]. 

These experiences suggest that the virus may remain on the farm not in the pigs but in the environment, and this environmental contamination might be one of the reasons that prevent the eradication of PCV2 in farms. In fact, more than a decade ago, Horlen et al. (2008) suggested that the reduction of the environmental viral load may be a key factor to consider when planning PCV2 control strategies [17]. In this regard, recent studies have tried to determine the epidemiological role of the environmental presence of PCV2, showing that the virus is widely distributed in the farm environment regardless of the production stage and that the viral loads are higher in PCV2 systemic disease situations [18,19,20,21,22].

In a previous study, we detected PCV2 DNA in the environment of different farm areas as well as in fomites and farm staff from non-vaccinated farrow-to-wean farms, revealing that weaning areas were the most contaminated facilities and highlighting a high PCV2 load in the environment of warehouses. These results suggest that these locations may act as points for maintaining and spreading the virus within farms [20]. However, as has already been mentioned, nowadays most farms perform a PCV2 vaccination programme, and no information about the PCV2 distribution and viral load in the environment of vaccinated farms is available. Thus, the identification of specific areas or fomites that can act as viral reservoirs in these farms is important to improve the current PCV2 control programs. Therefore, this study was performed to identify critical points for PCV2 contamination in the environment of farrow-to-wean farms which had been vaccinating piglets for years.

## 2. Materials and Methods

### 2.1. Included Farms, PCV2 Surveillance and Environmental Sampling

Four intensive farrow-to-wean farms from the region of Galicia (NW Spain) were included in the study. Briefly, all of them were of small size (less than 120 livestock units according to Spanish Real Decreto 306/2020 [23]), and they were selected because all of them had been carrying out a piglet vaccination scheme against PCV2 for at least seven years, without evidence or suspicion of PCV2 infection during that time. A more detailed description of the farms is supplied in Table 1. 

The four farms used the same commercial PCV2 vaccine (ORF2 expressed in baculovirus system) and were managed in 3-week batches, sending piglets to fattening farms upon finishing the weaning phase (at approximately 9–10 weeks of age). In addition, for all farms, the origin of replacement gilts and semen was the same. Although the number of buildings was different, all of them presented a similar distribution, including an office for administrative work and meeting farm visitors and a warehouse. Regarding external biosecurity, all farms gathered the requirements established by regulations, such as a perimeter fence, register book, distance to other farms, etc. As the main characteristics of internal biosecurity, all farms practiced an all-in/all-out system, applying a cleaning and disinfection protocol with a 1-week vacancy period between consecutive batches in farrowing and weaning rooms. No protocol for cleaning and disinfection of other facilities such as offices or warehouses was established, nor that evaluated the efficacy of that measures. The change of clothes and footwear before entering the farm and the use of exclusive workwear and work boots were compulsory in all of them. However, all farms practiced the change of clothes and boots among the different farm facilities, and none presented an established protocol to monitor the compliance of biosecurity measures.

A single visit was realized to each farm in 2019. During that visit the following activities were performed:

First, a total of 36 environmental samples from different surfaces and elements (corresponding to farrowing, weaning and gestation areas, warehouses, office, farm perimeter and farm staff) were taken using a previously reported swabbing method using sterile-cotton swabs of 11 mm in diameter [24]. Briefly, the protocol of swabbing consisted of moistening each swab with PBS-T (phosphate buffer saline with 0.05% of Tween 20, pH = 7.4) and swabbing the entire area of sampling; the specific swabbing protocol used for each type of sample is described in Table 2. Subsequently, swab heads were introduced in sterile screw-cap tubes by breaking the wooden stick of the swab and placed in a sample rack, which was maintained at room temperature in a foam box until processing in the laboratory in the following 24 h.

Second, the presence of PCV2 infection was assessed in piglets of approximately 8–9 weeks old in the weaning area: animals were visually evaluated in order to detect clinical signs compatible with PCV2 infections such as wasting, dyspnoea and/or skin pallor [1]; a total of 20 blood samples of these piglets (randomly taken for diagnostic purposes different from this study and provided by the veterinary practitioners of the farm) were also employed for PCV2 PCR analysis. The analysis of these 20 blood samples ensures the detection of at least one infected animal if the infection prevalence is at least 15% [25].

**Table 2:** Environmental samples and their swabbing protocol.

Environmental Sample	Swabbing Protocol (One Swab Per Sample)
Sow feeder ^1^, piglet resting area ^1^, piglet hopper ^2^, weaning pen wall ^2^, gestation sow hopper.	In eight different elements of each type, a 25 × 25 centimetre area per element [26].
Sow crate ^1^.	The surface of lateral and rearward lower bars of eight different crates.
Farrowing corridor ^1^, weaning pen floor ^2^, weaning corridor ^2^, gestation pen floor, gestation corridor, warehouse floor, office floor, parking area, farm main entrance, pig loading area.	100 steps were taken on each surface wearing polyethylene boot covers, and then both boot covers were swabbed as indicated previously [13]: in zigzag from the toe region to the heel.
Farrowing air fan ^1^, weaning air fan ^2^.	The surface of the fan blades or the protective grating
Delivery management toolbox ^1^.	The whole ventral external surface, 50% of the internal surface and all the syringes included in it.
Weaning pen railing ^2^.	1 m in length in zigzag.
Working utensils.	The handle of at least five different utensils (brushes, paddles…) for 10 s each.
Feed wagons, pressure washer, sorting panel, office tables/chairs.	50% of the surface.
Door handles.	The surface of the exterior and the interior office door handles.
Pens/Computer keyboard.	The surface of a computer keyboard or five different pens.
Carcass container	The whole surface of the winch.
Feed silo rungs.	The vertical and horizontal surfaces of five different rungs in at least two different silos.
Farmer hands and hair/hat.	Hands: the dorsal and ventral surface of the hands, including each finger and the ventral surface of each fingernail.
Workwear and streetwear.	The thorax area, the front and the back of each leg from the knee to the ankle, and the front and the back of each arm from the elbow to the wrist.
Work boots and street boots.	In zigzag from the toe region to the heel.
Farm staff vehicle.	A swab was rubbed on the door handle, the steering wheel, the gear shift lever, the handbrake, the pedals, the passenger seat and the dashboard

^1^ In the farrowing rooms with piglets older than 10 days. ^2^ In the weaning rooms with piglets older than 8 weeks.

### 2.2. Laboratory Analysis

Blood samples from each farm were pooled (four pools/farm); environmental samples were processed by adding 5 mL of PBS-T directly to each tube containing the swab head. The tubes were subsequently vortexed for 1 min and left to stand vertically for 15 min; 1 mL of supernatant from each tube was transferred to a sterile Eppendorf tube and kept at −20 °C until the DNA extraction was performed [27]. Briefly, both blood and environmental samples were processed using a commercial DNA extraction kit (High Pure PCR Template Preparation Kit, Roche Diagnostics GmbH, Mannheim, Germany) followed by real-time PCR (qPCR) analysis. For those environmental samples which tested negative, a second DNA extraction protocol was carried out using the commercial kit Nucleospin^®^ Soil (Macherey-Nagel GmbH & Co KG, Düren, Germany) following the manufacturer’s instruction. In all extractions, the starting volume was 200 μL of the swab eluate and the obtained DNA was collected in 100 μL of elution buffer. An exogenous internal control (EXOone EXIC, EXOPOL S.L., Zaragoza, Spain) was added to each environmental sample during the extraction in both protocols to identify possible qPCR inhibition and to evaluate the quality of DNA isolation. The collected DNA was kept at −20 °C until qPCR analysis.

The DNA samples were analysed using a commercial PCV2 qPCR kit (EXOone PCV2 oneMIX, EXOPOL S.L., Zaragoza, Spain), following the manufacturer’s instructions. A synthetic DNA positive control supplied with the kit was employed as the positive control and molecular-grade water was used as the negative control. A sample was considered positive when Ct ≤ 40 for the PCV2 detection channel according to the manufacturer’s instructions. Quantification (copies/swabs) was performed by the standard curve method, using serial ten-fold dilutions of the positive control (10^5^–10^1^ copies/µL) (Supplementary Materials). All qPCR reactions were run on an Applied Biosystems ABI Prism 7500 thermocycler (ThermoFisher Scientific, Waltham, MA, USA).

## 3. Results

None of the evaluated piglets at the end of the weaning stage showed clinical signs compatible with PCV2 infection or PCV2 viremia at the end of the weaning stage (0/16 positive pool samples). 

Regarding the environmental samples, the results of each sample from each farm are shown in Table 3. Briefly, a total of 22 out of 141 (15.60%) samples tested positive for PCV2 DNA. Only Farm C tested negative for PCV2 DNA. In the positive farms, the percentage of PCV2-contaminated samples per farm ranged from 19.44% to 22.22% (Table 3). 

The samples from the surfaces in direct contact with animals (farrowing, weaning and gestation areas) were negative (96.61%) except in the case of Farm A, which presented PCV2 contamination in the gestation area (Table 3). 

In contrast, PCV2 DNA was detected in non-animal facilities; in particular, the offices and the farm perimeter tested positive in three and two farms, which represented 37.50% and 21.05% of all the positive samples from all the studied farms, respectively. Similarly, the samples from the farm staff were positive in all the positive farms (Table 3), representing 25.00% of all positive samples, and a wide variation was observed regarding the viral load detected in them (5.89 × 10^2^–1.95 × 10^7^ copies/swab). Moreover, the samples from streetwear, street boots and/or vehicle accounted for 42.86% of the positive farm staff samples.

## 4. Discussion

The present study reveals that PCV2 DNA can be detected in the environment of long-term clinically healthy farrow-to-wean farms despite years of vaccination. Our most remarkable result is the detection of PCV2 DNA in different farm facilities without direct contact with the pigs, which contrasts with the scarce number of positive samples in the facilities housing animals. Although positive samples from these non-animal facilities (offices and farm perimeter, as well as farm staff’s streetwear and boots) were not the same type of sample in all the farms, the presence of viral contamination indicates a possible risk that these areas and fomites can be acting as viral reservoirs. Moreover, this observation, together with the absence of PCV2 contamination in animal housing areas and the negative PCR results from blood samples, might suggest an external origin for the detected PCV2 contamination. 

It must be noted that our aim here was to identify possible critical points for the accumulation of PCV2 in the environment, so this is the reason why we took different environmental samples from each facility. This allowed us to observe that PCV2 was not systematically present in the same samples across farms, but the viral DNA was detected in different samples from the same facility. Viral loads were generally low, so the role of this environmental contamination as a possible source of animal infection is uncertain; however, some samples showed high viral loads, such as the office floor in Farm D. Given that this is a cross-sectional study we cannot identify the time of contamination, so viral load and risk of infection may vary by time. In any case, our results indicate that high PCV2 contaminations can be detected in farm areas without any contact with the animals. It is worth mentioning that although the detection of PCV2 DNA does not imply viability, its mere presence should be interpreted as a potential risk for farms, due to the high resistance of this virus to adverse conditions [5,28].

As it has previously mentioned, visitors such as veterinarians, loading trucks, commercial agents, etc., can act as an external source of infection, since they can come contaminated from other farms or premises [13,14,15]. The presence of PCV2 DNA in the loading area from one farm may support this hypothesis. Although the result from this sample might respond to the existence of infected piglets that shed PCV2, it seems unlikely according to the obtained results, since these animals had been housed for almost two months in the weaning areas, where no PCV2 was detected. Thus, contamination from the truck cannot be discarded as the possible origin of the detected PCV2 DNA [29]. It should be noted that the same vehicle can be used first to transport adult pigs to the slaughterhouse, part of which can become infected and shed PCV2. Later, the vehicle can be used to carry piglets from farrow-to-wean farms, such as the ones included in the present study, to fattening ones. As a consequence, transportation vehicles play a well-known role in the transmission of the virus between farms [9,10,30,31,32]. Thus, this constant contact with external and uncontrollable farm events makes these areas the most critical points to eliminate the PCV2 from the farm environment. 

Regarding pig areas, PCV2 DNA was never detected in the environment of farrowing and weaning areas and was only found in the gestation area of a single farm, which indicates a possible infection in the breeding population. Although we did not analyse animals from the breeding population, according to previous information, adult sows are unlikely to be infected in farms that vaccinate piglets, but replacement gilts may arrive infected from their origin farm [33,34,35]. Thus, it seems likely that in the farm with positive results in the gestation area, some animals (replacement gilts/sows) may have been infected and shedding PCV2, therefore contributing to contaminating the environment of these facilities.

The existence of PCV2 contamination in farm facilities might entail a source of infection for susceptible animals, contributing to re-infections and limiting the possibility of achieving an eventual eradication of PCV2. Regarding this, the contamination observed in samples taken from the farm staff supports their probable role as viral disseminators among the different farm facilities, regardless of the origin of the source of contamination. For example, if facilities such as offices or warehouses become contaminated with PCV2 from an external source, the farm staff can facilitate the spread of the virus to animal dependencies [13,36]. Although biosecurity measures, such as the mandatory change of boots and clothes before entering the farm are very frequent, our results suggest that they may not offer the expected efficacy; moreover, the lack of strict protocols which monitor the compliance and the effectiveness of these measures agrees with what has been stated by other authors [37]. Therefore, the implementation of a specific biosecurity protocol for working in pens or dependencies used to house sick pigs would be advisable.

Despite PCV2 vaccination programmes having been effective in decreasing the prevalence of the disease and its associated losses, achieving eradication seems to be more complex since infections usually tend to re-emerge when the vaccination programmes cease. Our results evidence the presence of PCV2 in long-term vaccinated swine farms and point out the usefulness of examining surface samples to detect the virus, thus contributing to the knowledge of the infection dynamics. However, provided that the number of farms included in this study is low, the extrapolation of the results to a larger population may not be completely reliable, since exposure risks vary between farms due to the differences in management practices and, furthermore, infection levels can change over time. However, the detection of viral contamination in some facilities without animals is noteworthy, therefore, these areas must be considered in control programmes since they could act as viral reservoirs.

Even so, the viability of the detected particles of PCV2 DNA, as well as the assessment of the contamination flows, and the potential mechanisms of viral persistence are some aspects that should be considered in further studies to achieve a better characterization of the PCV2 infection dynamics and to identify the critical points for a future eradication.

## 5. Conclusions

Although PCV2 vaccination has been performed for years in the studied farms, the presence of PCV2 DNA in environmental samples could indicate that the virus is still present in certain specific points of the farms. PCV2 contamination was almost inexistent in the animal housing facilities but was more frequent in those without animals, such as the offices, the farm perimeter or the farm staff’s street elements, indicating their potential role as viral. As the presence of PCV2 in these critical points could imply a risk of the re-emergence of the disease if vaccination ceases, our results point out the need for regular and effective cleaning and disinfection procedures in these facilities and elements in order to minimize the risk of reinfection from environmental sources.

## Figures and Tables

**Table 1:** Characteristics of the farms.

Farm	A	B	C	D
Number of sows	360	200	300	300
Number of buildings	3	1	1	1
Number of farrowing rooms	7	6	6	6
Number of weaning rooms	6	4	6	7
Number of gestation rooms	1	1	1	1
PCV2 vaccination strategy	Only piglets at 4 weeks of age	Only piglets at 3 weeks of age	Only piglets at 3 weeks of age	Only piglets at 3 weeks of age
Years of vaccinating against PCV2	7	7	8	8
PRRS infection	Negative	Negative	Negative	Positive

**Table 3:** Number of PCV2 copies/swabs in each environmental sample in each farm.

Farm Facility	Sample	Farm N° of Environmental Samples (% of PCV2 Positive Samples)			
		A 33 (21.21%)	B 36 (22.22%)	C 36 (0%)	D 36 (19.44%)
Farrowing area (piglets older than 10 days)	Sow feeder	-	-	-	-
	Sow crate	-	-	-	-
	Piglet resting area	-	-	-	-
	Corridor	-	-	-	-
	Air fan	-	-	-	-
	Delivery management toolbox	Not sampled *	-	-	-
Weaning area (piglets older than 8 weeks)	Piglet hopper	-	-	-	-
	Pen wall	-	-	-	-
	Pen floor	-	-	-	-
	Corridor	-	-	-	-
	Pen railing	-	-	-	-
	Air fan	-	-	-	-
Gestation area	Sow hopper	-	-	-	-
	Pen floor	2.63 × 10^3^	-	-	-
	Corridor	1.74 × 10^5^	-	-	-
Warehouses	Floor	9.55 × 10^3^	-	-	5.01 × 10^4^
	Working utensils	-	-	-	-
	Feed wagons	1.02 × 10^3^	-	-	-
	Pressure washer	Not sampled *	-	-	-
	Sorting panels	-	-	-	-
Office	Floor	-	7.94 × 10^2^	-	1.20 × 10^7^
	Door handles	1.45 × 10^3^	9.77 × 10^2^	-	-
	Pens/Computer keyboard	-	7.08 × 10^2^	-	-
	Tables/Chairs	-	9.12 × 10^3^	-	-
Farm perimeter	Parking area	-	-	-	-
	Farm main entrance	-	-	-	8.13 × 10^2^
	Pig loading area	Not sampled *	-	-	1.51 × 10^3^
	Carcass container	-	1.10 × 10^3^	-	-
	Feed silo rungs	-	6.46 × 10^2^	-	-
Farm staff	Hands	-	1.02 × 10^3^	-	-
	Hair/hat	-	-	-	-
	Workwear	-	9.33 × 10^3^	-	-
	Work boots	1.66 × 10^4^	-	-	1.95 × 10^7^
	Streetwear	-	-	-	5.89 × 10^2^
	Street boots	7.76 × 10^2^	-	-	1.95 × 10^5^
	Farm staff vehicle	-	-	-	-

- Indicates a negative result. * Indicates that this element was not available on the farm.

## Data Availability

The data of this study are available from the corresponding author upon reasonable request.

## Supplementary Materials

The following supporting information can be downloaded at: https://www.mdpi.com/article/10.3390/ani12243515/s1, qPCR standard curve parameters and cycle threshold values for its calculation.
