# Supplementary material for: Presence of Porcine Circovirus Type 2 in the Environment of Farm Facilities without Pigs in Long Term-Vaccinated Farrow-to-Wean Farms
Source: Animals (Basel). 2022 Dec 13;12(24):3515. doi: 10.3390/ani12243515 (PMC9774950; doi:10.3390/ani12243515)
Supplement: Supplementary file 1 [file animals-12-03515-s001.zip › animals-2023626-supplementary.pdf]

SUPPLEMENTARY MATERIAL

qPCR standard curve parameters and cycle threshold values for its calculation

Standard curve: $CT = 40.141 - 3.405 * log10\ [PCV2\ copies]$

$\mathbf{R^2} > 0.99$

Efficiency = 96.642 %

**PCV2 control positive dilutions**	**qPCR cycle threshold (CT) values employed for standard curve calculation**
5 $x10^1$ copies/rxn	34.30 and 34.41
5 $x10^2$ copies/rxn	30.96, 30.99 and 31.02
5 $x10^3$ copies/rxn	27.42 and 27.50
5 $x10^4$ copies/rxn	24.09 and 24.21
5 $x10^5$ copies/rxn	20.72 and 20.79
